# Supplementary material for: The Environment-Dependent Regulatory Landscape of the E. coli Genome
Source: ArXiv. 2025 May 13:arXiv:2505.08764v1. Preprint. [Version 1] (PMC12133099)
Supplement: Supplement 1 [file NIHPP2505.08764v1-supplement-1.pdf]

Supplemental Information for: The Environment-Dependent Regulatory Landscape of the *E. coli* Genome

Tom Röschinger [1], Heun Jin Lee [2], Rosalind Wenshan Pan [1], Grace Solini [1], Kian Faizi [1], Baiyi Quan [3], Tsui-Fen Chou [1, 3], Madhav Mani [4], Stephen Quake [5, 6], and Rob Phillips [1, 7, +]

[1] *Division of Biology and Biological Engineering, California Institute of Technology, Pasadena, CA 91125, USA*
[2] *Department of Applied Physics, California Institute of Technology, Pasadena, CA 91125, USA*
[3] *Proteome Exploration Laboratory, Beckman Institute, California Institute of Technology, Pasadena, CA 91125, USA*
[4] *Department of Engineering Sciences and Applied Mathematics, Northwestern University, Evanston, IL 60208, USA*
[5] *Department of Bioengineering, Stanford University, Stanford, CA 94305, USA*
[6] *Department of Applied Physics, Stanford University, Stanford, CA 94305, USA*
[7] *Department of Physics, California Institute of Technology, Pasadena, CA 91125, USA*
[+] *Correspondence: tom@caltech.edu, phillips@pboc.caltech.edu*

Contents

S1 Existing methods for dissection of gene regulation in bacteria **44**

S2 Theory of the experiment **47**

S2.1 Expression Shifts . . . 47

S2.2 Mutual Information . . . 48

S2.3 Identification of Binding Sites . . . 48

S2.4 Computational identification of transcription factor binding partner using Tomtom . . 53

S3 Compendium of All Promoters in Our Study **55**

S4 SI Tables and Figures **57**

S1 Existing methods for dissection of gene regulation in bacteria

A huge effort has been expended in uncovering how genes in *E. coli* are regulated [4, 22, 24, 28, 105–112]. For much of the history of modern molecular biology, genes were usually studied on a one-by-one basis due to the lack of high throughput methods. This led to major success stories including insights into the lysis-lysogeny decision in bacteriophage lambda, discoveries on how bacteria use different carbon sources including lactose, galactose and arabinose, insights into the role of DNA looping and a myriad of other examples. Over the past few decades, a variety of high-throughput methods have re-enlivened the subject by enabling the identification of many binding sites for a single

transcription factor in one experiment, or identifying binding sites for all transcription factors without knowing the identity of the transcription factors specifically binding those sites. Here we provide an overview of some examples of previous work to give a sense of where our own efforts fit into this enormous subject, highlighting both the successes and open questions resulting from previous work. We begin by discussing in vitro approaches in which DNA and proteins are interrogated outside of their natural cellular environments. Much has been learned from these approaches. Then, we turn to the analysis of in vivo methods which attempt to capture DNA-protein interactions in the context of living cells.

- SELEX: Systematic evolution of ligands by exponential enrichment (SELEX) was developed in 1990 [113] with the purpose of identifying *in vitro* which DNA sequence or ligand a protein binds to. The method uses a library of synthesized DNA that is incubated with purified proteins. Unbound DNA is removed and bound DNA is eluted from the protein and subsequently amplified. This process is repeated over multiple rounds to find DNA with high binding affinity to the protein. The level of specificity of the DNA obtained in these experiments can be tuned by choosing a different number of cycles. Once a DNA sequence with high affinity for the protein is identified, the genome of interest can be scanned for potential transcription factor binding sites by looking for sequence similarity between the genomic DNA and the SELEX DNA. In the context of *E. coli*, binding sites for hundreds of transcription factors have been identified genome wide [57].
- PBM: Protein-binding microarrays (PBM) use a large array of synthesized DNA oligonucleotides that are fused to a surface. Binding of purified protein to the DNA oligonucleotides is measured by fluorescence microscopy of tagged proteins. PBMs are able to better detect less specific binding sites for TFs than SELEX [114]. PBMs have been used to identify the motifs for more than 1000 transcription factors [115].
- DAP-Seq: One modification to *in vitro* binding assays that has been useful is to choose genomic DNA as template instead of synthesized DNA. This approach is the basis of DNA affinity purification sequencing (DAP-Seq) [116, 117]. One of the advantages of using genomic DNA is that such DNA maintains chemical modifications to the DNA such as methylation and reveals that such methylation can be important for DNA - TF interactions.
- ChIP-Seq: Chromatin immunoprecipitation with sequencing (ChIP-Seq) is one of the most commonly used methods to identify binding sites for TFs in the *in vivo* setting. Identifying binding sites *in vivo* is beneficial as potential co-factors and enzymes modifying the conformation of the TF are present if the correct growth condition is chosen. In the first iterations of the method the resolution of identified binding sites was low, however, the use of endonucleases in ChIPexo-Seq [118] lead to higher accuracy. To pull down TFs that are crosslinked to DNA, the TF needs to be modified with a tag, e.g., His-tag [119], or antibodies against the TF need to be available, which can limit the throughput and often means that only one or a few TFs can be studied at the same time. However, binding sites across the entire genome can be found in a single experiment, giving high throughput on this axis. There have been drastic differences in the number of binding sites identified for certain TFs between ChIP-Seq and SELEX [119]. ChIP-Seq has been used to discover a variety of different DNA-Protein interactions, such as the RpoS regulon in *E. coli* [119], the PhoB regulon [120], nucleoid organization by H-NS and MukBEF [121], genome wide binding of CRP (using DNA microarrays) [122] and binding of Sigma70 [123]. In a recent study, ChIp-Seq was performed on 139 TFs for cells grown in minimal media with glycerol [124].

- DNAse footprinting: In contrast to ChIP-Seq, DNAse footprinting does not require a pull down on a specific TF. This allows for the discovery of binding for all DNA binding proteins across the entire genome at the same time, but also comes with the loss of the identity of the protein binding to each site. This method has been combined with RNAP occupancy studies to verify the function of identified binding sites [12, 125].
- MPRA: Massively-parallel reporter assays are one of the signature recent achievements in high-throughput approaches for dissecting promoter function. The method is based upon creating large libraries of genetic variants and measuring their function in parallel. Urtecho et al. use genome-integrated massively parallel reporter assays to catalog and characterize promoters throughout the *E. coli* genome [96]. This approach has been used impressively in *E. coli* to explore not only binding sites, but also ribosomal binding site sequences [126], etc.
- Sort-Seq: In Sort-Seq [7, 29] binding of transcription factors is identified by mutating bases in the vicinity of a transcription start site and measuring expression of a downstream reporter gene using fluorescence activated cell sorting (FACS), followed by DNA sequencing of the mutated promoter variants. By identifying bases where a mutation leads to a large change in expression putative binding sites are discovered. The identity of the transcription factor binding to these sites is then identified by DNA chromotography and mass spectrometry. This approach not only identifies binding of transcription factors, but also shows that the binding is functional, i.e., binding of the transcription factor effects expression of a gene. It has been found that binding sites that have been identified in vitro do not necessarily imply that the binding has regulatory function [120].

All of these methods have been used to gain insights into the regulatory landscape of *E. coli* and other organisms. Databases such as EcoCyc [52] and RegulonDB [127] contain a large number of annotated binding sites. Our goal is to use data from these methods as well as approaches in our paper to provide a systematic, rigerous and complete description of all promoters in *E. coli* with standardized annotations in databases that can be used for e.g. phylogenetic modeling and building blocks for synthetic biology. In particular, as shown in several of the figures in the paper, in those cases where we are able to find putative binding sites and identify the TFs that bind to those sites, the aim is to be able to go from a promoter of unknown regulatory architecture all the way to an environment-dependent regulatory architecture including energy matrices describing transcription factor binding and statistical mechanical models of the input-output response of the promoter of interest as a function of key regulatory knobs such as DNA-TF binding site strength, TF copy number and effector concentration. Beyond that, the aim is for all of these disparate sources to come together to make excellent databases such as EcoCyc [52] and RegulonDB [127] a more complete and internally consistent source as a basis for rigorous understanding of the physiology and evolution of *E. coli* and that will serve as a template for how to structure such databases for other organisms.

Sequence	DNA counts	RNA counts
ACTA	5	23
ATTA	5	3
CCTG	11	11
TAGA	12	3
GCGC	2	0
ACCA	8	7
AGTA	7	3

Table S1. Synthetic dataset used to explain the logic of creating summary statistics. Hypothetical dataset where for each sequence there is an associated count from DNA sequencing and a count from RNA sequencing. For this hypothetical case, the tiny wild-type "sequence" is ACTA.

S2 Theory of the experiment

For each promoter-growth condition pair we produce a unique dataset consisting of counts of both DNA and RNA for each barcode. An example dataset is shown in Table S1. To identify potential binding sites in the sequence, we are looking for positions in the sequence of interest that lead to a high change in expression of the reporter when the base is mutated. We hypothesize that this coupling between sequence and expression indicates that the binding of a regulatory factor has been modified. There are multiple ways of displaying the connection between the identity of each base and the level of associated with such sequence changes. Ultimately, we have opted to use "summary statistics" that take as input the sequencing reads and result in output of some basepair by basepair picture of the importance of a given base to the level of expression. In particular, here we use two such summary statistics, namely, expression shifts and mutual information as a way to generate hypotheses for binding site positions and sequences. A detailed discussion of both quantities can be found in [54]. Here, we give a brief reminder of these two summary statistics to make our approach self-contained, though the reader can see that reference for more details.

S2.1 Expression Shifts

The expression shift summary statistic is an average way of determining how much the gene expression will be changed if the base at site i is changed from its wild type value to one of the three other alternatives. Expression shifts can be calculated directly from the data, and measure directly how much the expression is changed given a mutation to a specifc base at each position. The result is a $4 \times L$ matrix, where L is the length of the promoter sequence. Specifically, for a dataset where each sequence i is associated with a measure for expression c_i the value in the expression shift matrix at position l corresponding to base b is given by

$$\Delta s_{b,l} = \begin{cases} \frac{1}{n}\sum_{i=1}^{n} \xi_{i,l}\left(\frac{c_i}{\langle c\rangle} - 1\right), & \text{where} \quad \langle c\rangle = \frac{1}{n}\sum_{i=1}^{n} c_i, \quad \text{if } b \text{ is mutated} \\ 0, & \text{if } b \text{ is wild type} \end{cases} \tag{S1}$$

where $\xi_{i,l} = 1$ if the base at position l in the i-th promoter variant corresponds to base identity b and $\xi_{i,l} = 0$ otherwise. For our experiments, the measure of expression c_i is the ratio of RNA to DNA barcode counts. The same measure can be applied to Sort-Seq datasets, where the measure of

expression is the expression bin the sequence was assigned to. This matrix form of the expression shift allows us to capture how mutations in specific bases affect the promoter change in expression.

S2.2 Mutual Information

As noted above, a second useful summary statistic is the information footprint. Details for how to go from sequence reads to this summary statistic can be found in [54]. This way of displaying how mutations change expression of the reporter gene is achieved by computing the mutual information between the identity of a given base and the level of gene expression. High mutual information indicates that the identity of the base is significant in governing the level of expression and leads to the hypothesis that that base is part of a binding site, for example. However, as we showed in Figure 2, not only does the information footprint reveal binding sites that had not been seen before, but also sometimes hides the existence of mutations that created new transcription start sites. Ultimately, the information footprint is one of many possible summary statistics for trying to tame the enormous datasets, and needs to be used judiciously.

From the data we can compute the ratio of reads that contain a mutation at each position for both DNA and RNA reads, $p(m, \mu)$, where m indicates the base is wild-type ($m = 0$) or mutated ($m = 1$), and μ indicates if the read belongs to DNA ($\mu = 0$) or RNA ($\mu = 1$). Then, we compute mutual information at position i as

$$I_i = \sum_{m=0}^{1} \sum_{\mu=0}^{1} p(m, \mu) \log_2 \left(\frac{p(m, \mu)}{p(m)p(\mu)} \right), \tag{S2}$$

where $p(\mu)$ and $p(m)$ are the marginal distributions of $p(m, \mu)$. In the example dataset introduced in Table S1 for the purposes of explaining notation and the algorithm, there are a total of 50 DNA counts and 50 RNA counts. In the first position, the wild-type base is A, and 25 DNA reads contain the wild-type base, hence we compute $p(0, 0) = 0.25$, while 36 RNA reads contain the wild-type base, thus $p(0, 1) = 0.36$. 25 DNA reads contain a mutation in the first base, which gives $p(1, 0) = 0.25$, and 14 RNA reads contain a mutation in the first base, therefore, $p(1, 1) = 0.14$. Using these values, using equation S2 we can compute mutual information for the first base to be $I_1 = 0.037$ bits. At this point in time, the meaning of an absolute value is still unclear, and we can only compare the value of mutual information at one position to the values at other positions in the same promoter.

S2.3 Identification of Binding Sites

Once an information footprint is computed, one has to identify where binding sites are. For a handful of footprints this can be done by hand, however, for the scale of this experiment when on the order of 10000 unique footprints are obtained, an unbiased and automated method to detect binding sites from the data is needed. We have explored multiple different methods which are discussed below.

S2.3.1 Triaging

At first, we put each footprint into one of three categories: 1. Footprints containing putative binding sites for transcription factors and/or sigma factors 2. One single mutation in the promoter sequence leads to high expression from a transcription start site that is different from the annotated transcription start site, a phenomena we call *de novo promoters* 3. The footprint is not distinguishable

from noise. The mutual information computed from equation S2 is at base-pair resolution. To smoothen the footprint and make it easier to identify peaks, we use a sliding gaussian kernel across each footprint. An important quantity for our classification of footprints is the coefficient of variation (CV) of mutual information across the promoter. A footprint with distinct peaks compared to a noise background has a large CV compared to a footprint with lots of fluctuations, as shown in Figure S1(A). To estimate a noise floor for our dataset, we use a shuffling approach. We take our datasets (measurements of promoters in growth conditions), and assign sequencing counts to random mutated promoter variant that is different from the sequence the counts were identified initially. This removes the measured correlation between base identity and effect of the mutation. We perform this shuffle 100 times per promoter-growth condition pair, and for each iteration the footprints are smoothed using the gaussian kernel and the CV is computed. All resulting CV values are pooled, and we compute a 95% confidence interval for the distribution, where we choose the upper limit of the interval as the noise threshold, which turns out to be 0.75, as shown in Figure S1(B). This allows us to distinguish measurements that are dominated by noise from footprints that contain a distinguishable signal. To identify if a footprint contains joint regions of high mutual information, a sign of a binding site, or a single mutation that leads to activity from the promoter, we look at how the smoothing of the footprint changes the CV across the positions. We simulate footprints with varying number of neighboring positions that have high mutual information compared to a noisy background, and compute the CV before and after smoothing of these footprints. We find that there is a distinct group of datasets that follow the trend for a single position with high mutual information, as can be seen by the blue line in Figure S1(C). This allows us to separate the datasets between footprints that have putative binding sites, and footprints with single positions of high mutual information. Figure S1(D) shows the thresholds and example footprints for the *araB* promoter. We then use a Hidden Markov model to identify binding sites for promoters in the first class and the computational model by LaFleur et al. [56] to look for putative new transcription start sites in promoters with single mutations of high mutual information, both cases are discussed in detail below.

S2.3.2 Hidden Markov Model

Once a sequence is identified as containing binding sites, we use a two state Hidden Markov Model to distinguish binding sites from positions that do not contain binding sites. Hidden Markov models are often used for sequence analysis [55], and here we present an application specific to the nature of our experiment and datasets. The two hidden states in the model are binding sites and background, where we assume that binding sites contain significantly higher mutual information that noisy background. Positions that do not contain binding sites have non-zero mutual information due to experimental noise but also due to the nature of our mutated library, where each sequence contains on average 16 mutations (160 bases at a 0.1 mutation rate). Hence, mutations in and outside binding sites occur at the same time. Since we are using about 1500 mutated sequences per promoter, we are using only a tiny fraction of all possible mutated sequences. The observable in the model is the mutual information in the footprints, which are treated like time-series, in the sense that each position can be interpreted as a subsequent "time point". The transition probability matrix in the model captures the fact that binding sites are not at single base pairs, but instead usually made out of groups of 10-20 bases at a time. We use gaussian distributions to parameterize the emission probabilities, and use the "hmmlearn" Python package to fit and evaluate the models. For each information footprint we fit a individual model, repeating the process 10 times to exlude possible diverging runs. From the 10 separately fit models, we choose the one that produces the highest log-likelihood when the sequence

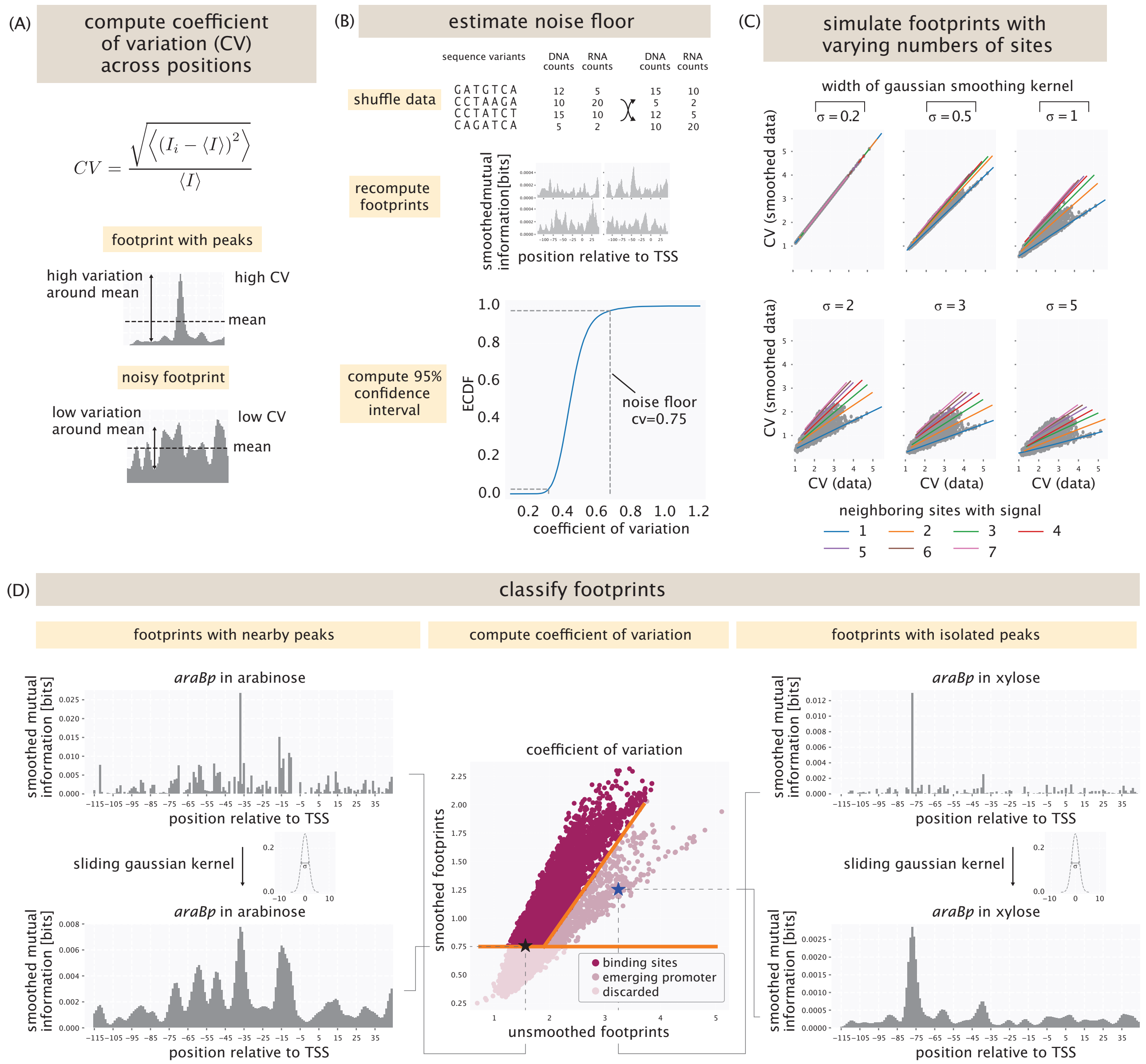

Figure S1. Triaging of the data into different classes. (A) The coeffiecent of varation of mutual information across positions in the footprint is a proxy for noise. (B) The noise floor of measurements can be estimated by shuffling datasets and recomputing footprints and coeffiecents of variation. (C) Datasets follow different trends under smoothing depending on the number of positions with high mutual information. Footprints with single positions of high mutual information separate from the rest of the data. (D) Based on the noise floor and the distinct behavior of footprints with single positions of importance, thresholds are drawn in the plane of coefficient of variation before and after smoothing of the data. Shown are examples for *araB*, which has activator binding sites when cells are grown in arabinose, but not when grown in xylose. In that case, a single mutation upstream of the annotated TSS leads to a new active TSS emerging.

is evaluated using the model. The information footprint itself is unsigned and hence, does not contain information about binding sites being repressor-like or activator-like. When a region of the promoter is identified as binding site, we look at the expression shift to identify the sign of change in expression when the site is occupied. In Figure S2 we show the result of the Hidden Markov Model for the *araB* promoter for growth in arabinose, and the identified binding sites, which overlap with the annotated activator sites for AraC. In the expression shift we can see that within the binding sites, nearly every mutation leads to a decrease in expression, showing how mutations in the binding site weaken binding of the activator and decrease expression.

As quality control, fit the model both for the forward sequence, but also the reverse sequence, to ensure consistency. Examples are shown for the *araB* promoter for growth with arabinose and the *tisB* promoter for growth in glucose. Another important parameter is the width of the gaussian kernel used for smoothing. If the smoothing kernel is too narrow, binding sites are not identified as continuous units and many single positions outside actual binding sites are identified as binding sites by the model. If the kernel is too wide, binding sites merge and we lose the resolution to distinguish neighboring binding sites, as shown in Figure S2. Thus, we choose a kernel width of $\sigma = 2$ for our analysis.

S2.3.3 *De novo* promoters

As seen in the main text, one of the key and surprising outcomes of our experiments was the emergence of new transcription start sites based only upon a key driver mutation. This phenomenon was discovered in earlier experiments by the Gore Lab [82]. The presence of such results in our experiments led us to develop a systematic approach for identifying these new transcriptional start sites as shown in Figure S3.

The most useful way of visualizing the emergence of new promoters in our data is using the summary statistic we call the expression shift matrix. In the left panel of Figure S3 we show an example of such an expression shift matrix in the context of the *araBp* promoter when grown in xylose. As seen in the figure, there is a mutation just to the left of the -75 position in which a G is replaced by an A and for which there is a very large resulting shift in the expression. To investigate if this mutation indeed led to formation of a new transcription start site, we split the dataset into two groups, one that harbors the mutation at this site, and a second group that has the wild type base at that site. At this point in the procedure, we invoke bioinformatics in the form of a model from the Salis lab for predicting transcription rates from putative promoter sequences [56]. As seen in the middle panel of the figure, our approach is to slide along the promoter region in single base pair increments, where for each position, we extract a block of sequence and feed into into the Salis Lab transcription rate calculator. The result is a predicted transcription rate for every possible transcription start site for each sequence in both groups. To summarize the outcomes, we compute the average transcription rate per transcription start site across all sequences within each group. The average predicted transcription rate is then plotted as a function of the position of our query block of sequence as shown in the right panel of the figure. As is evident in the figure, there is significant enhancement in the predicted transcription rate that corresponds precisely with the presence of the mutation of interest. As noted in the main text, such mutation are precisely in the -10 region of a new promoter.

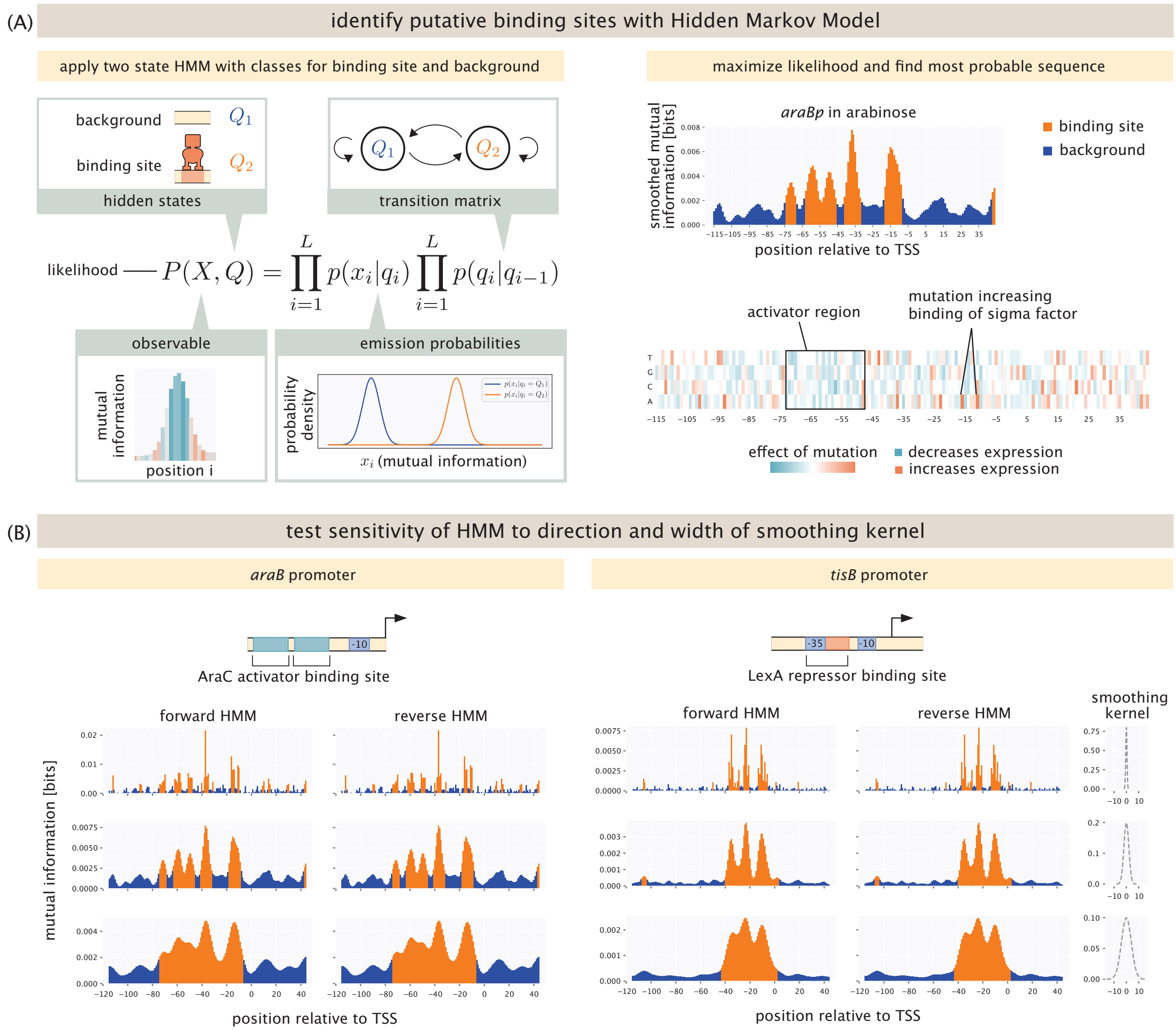

Figure S2. Using Hidden Markov Models to find binding sites in information footprints. (A) There are two hidden states associated with each basepair. We identify a given basepair either as being part of a binding site or not (these sites are labeled as background). The observable used is the mutual information per position. To maximize the likelihood, a transition matrix is fit which gives the prior probability that the $i+1^{th}$ position is a certain state given the state of the ith position. Emission probabilities describe how likely it is to find a value for mutual information in a binding site or in background. Once the model is fit, the sequence of states which returns the maximum likelihood is returned, and binding sites are identified. The expression shift matrix is used to find if the binding site is activator-like or repressor-like. (B) Test of the Sensitivity of the Hidden Markov model by examining the consistency of the analysis when going from right to left rather than left to right and as a function of the width of the smoothing kernel.

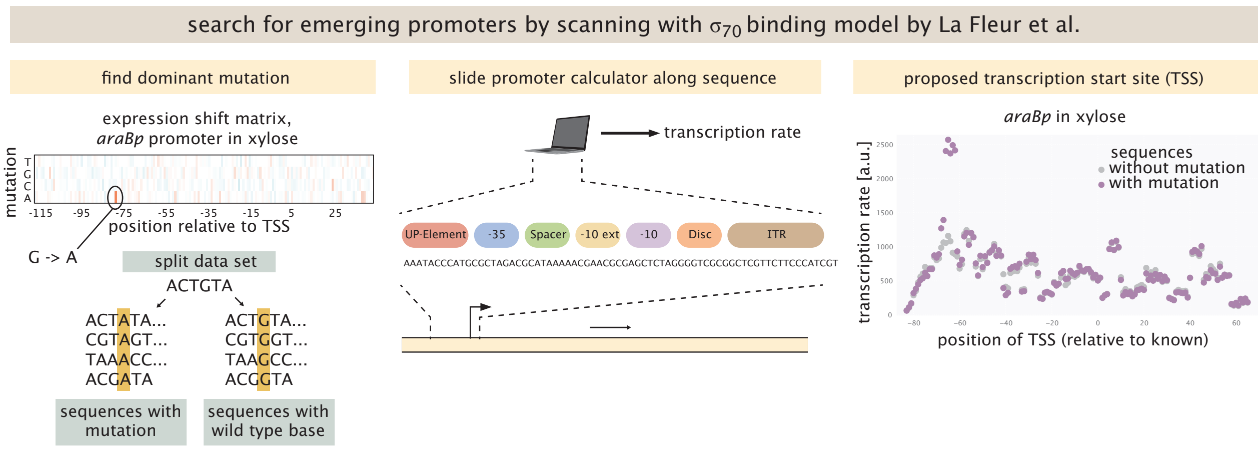

Figure S3. Using a computational model to identify putative transcription start sites. When a promoter is identified to contain a putative new transcription start site, the mutation with the largest expression shift is identified. The sequences for this promoter are split into a group that contain that mutation and all sequences that do not carry that mutation. The model of LaFleur et al. [56] is used to predict a transcription rate for every possible transcription start site in the sequence. The model takes different elements of the σ^{70} factor into account. We then visualize the predicted transcription rate for every possible transcription start site and look for a *de novo* promoter.

S2.4 Computational identification of transcription factor binding partner using Tomtom

After generating hypotheses about putative binding sites in each promoter region, our next task is to identify which transcription factor binds to each of these binding sites. To do this, one approach is by comparing our putative binding sites with datasets of transcription factor binding sites that have been previously reported in the literature. If a query binding site shares large similarities with a known binding site, we can then hypothesize that the query binding site likely has the same binding partner as that known binding site.

To perform this motif comparison, we use the algorithm Tomtom, which identifies which known binding motifs have statistically significant similarities with a given query binding site sequence. In particular, Tomtom searched for hits for both the forward sequences of the known binding motifs as well as the reverse complement of the known binding motifs. For each query-target pair, Tomtom produces an optimal alignment and scores the overlapping region using a chosen distance metric. In our case, we choose to use Euclidean distance. Tomtom then computes a p-value under the null hypothesis that the two motifs are drawn independently from the same probability distribution, and reports a corresponding q-value that estimates the false discovery rate. We retain only motif matches that exceed a minimum q-value of 0.5 and a minimum overlap length of 15.

In this analysis, the known binding sites are downloaded from RegulonDB and Ecocyc. RegulonDB contains 4897 binding sites and EcoCyc contains 2781 binding sites. There are many duplicated or overlapping binding sites within each database and between the two databases. We first removed the duplicated entries and combined regions with more than 90% overlap within RegulonDB and EcoCyc

databases separately. This resulted in 3319 binding sites in RegulonDB and 2763 binding sites in EcoCyc. Finally, we consolidated the two databases by merging sequences with over 90% overlap, and we have a final list of 4426 binding sites across the two databases.

After a transcription factor binding partner is found at a particular binding site, we use the sequence of that particular binding site as the query sequence for Tomtom and search it against all target sequences with a minimum q-value of 1. This allows us to generate plots to plot the distribution of transcription factor binding p-values and q-values at that particular site and visualize the p-value and q-value of the transcription factor that is determined as a hit relative the p-values and q-values of other transcription factors.

S3 Compendium of All Promoters in Our Study

In this section we give a description of our compendium of every promoter we studied in our experiments. In some ways, the compendium is an organized but informal notebook of all the genes we considered as an aid to those who are interested in a summary of what was found for each promoter under different conditions. In another sense, the compendium is a visual summary of the huge dataset that emerged from our study. We also refer interested readers to the online resources http://rpdata.caltech.edu/data/interactive_footprints.html and http://rpdata.caltech.edu/data/all_data.pdf.

For each promoter, we show a cartoon figure of annotated binding sites and the conditions they were identified in. To set both notation and to define the visual icons used in the paper and in this section, Figure S4(A) presents an example which describes our color scheme for labeling activator binding sites, repressor binding sites, binding sites of dual function and binding sites for sigma factors. Each of the figures in our compendium also shows the information footprints that reveal our results for these promoters in relevant growth conditions. Figure S4(B) gives an example of the way we will show our information footprints throughout the compendium. Figure S4(C) shows one of our most useful ways of visualizing our data with a compact figure that shows for each growth condition of interest the regions of the promoter in which something interesting was found. In this figure, we show both the emergence of *de novo* transcription start sites as well as putative binding sites.

In this long compendium, for each promoter, we briefly review the literature to identify binding sites that have been previously annotated and judge if the binding sites were identified in our experiments. Previously annotated binding sites from the literature can escape detection in our experiments for a variety of reasons, e.g., the growth conditions in which the original discovery of the binding site was made are not included in the set of conditions that we used. As a result, the transcription factor of interest might not bind at all in our experiments. It is also possible that binding sites were identified in previous work using strains or conditions in which there was over-expression of a transcription factor. Since our *E. coli* strain is not over-expressing any transcription factors, the effect of lower transcription factor copy numbers might result in circumstances in which binding is not measurable. Our own earlier theory-experiment dialogue has used transcription factor copy number and plasmid copy number as a key tuning variable that can lead to 1000-fold changes in gene expression, so it is clear that copy number effects are crucial and can make the difference between a measurable effect and not [39, 53]. Transcription factors that regulate their promoter by interacting with other binding sites through action at a distance, e.g. by DNA looping, are also hard to identify, since the additional binding sites required for regulation might not be included in our reporter construct, as we only study a 160 base pair region around the transcription start site.

We are cognizant that this is an extremely long compendium. Our reason for including it is driven by a philosophical conundrum of this era of big data in biology. How do we find ways to talk about our data other than in giant spreadsheets? We decided here to make the specific details of every promoter available for those who might have interest in a given promoter. The full compendium can be found under this link: http://rpdata.caltech.edu/data/reg-seq_compendium.pdf.

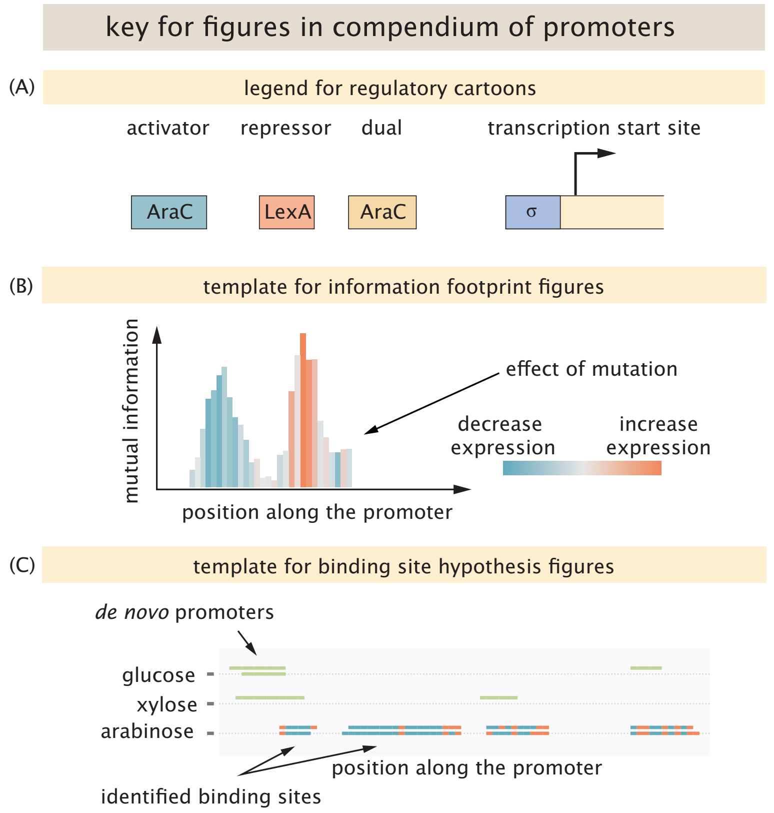

Figure S4. Key for regulatory cartoons and information footprints. (A) Binding sites for activators are shown using teal boxes. Repressor binding sites are shown using orange boxes. -10 and -35 regions of σ^{70} are shown in blue boxes. Transcription start sites are indicated by arrows. (B) Information footprints are displayed as mutual information per position. For easier interpretation, each position is averaged with its two neighbors on each side. Each position is colored by the average expression shift at that position, indicating if a mutation decreases expression on average (shown in teal, indicative of a binding site for an activator) or increases expression on average (shown in orange, indicative of a repressor). (C) For each promoter where regions in information footprints were identified as putative binding sites, we show a figure that highlights such regions. Each base is colored by being either repressor-like (increasing expression when mutated), in red, or activator-like (decreasing expression when mutated), in blue. If a footprint was classified to contain a *de novo* promoter, the bases are highlighted in green instead.

S4 SI Tables and Figures

Growth Condition of Lysate	Final Concentration of Supplement(s) Added
M9-Glucose	0.5% Glucose, 1 mM cAMP
M9-Arabinose	0.5% Arabinose, 1 mM cAMP
M9-Xylose	0.5% Xylose
LB	None Added
Stationary Phase (1d)	1 mM cAMP
Leucine	10 mM Leucine
Phenazine Methosulfate	100 µM Phenazine Methosulfate
2,2 Dipyridyl	5 mM 2,2 Dipyridyl
Gentamicin	5 mg/l Gentamicin
Copper Sulfate	2 mM Copper Sulfate
Heat Shock	None added
H_2O_2	2.5 mM H_2O_2
High Osmolarity (LB + 750 mM NaCl)	200 mM NaCl, 150 mM KC1, 1 mM cAMP

Table S2. Growth Conditions for lysates used for mass spectrometry and associated supplements added. Notes: A supplement of 1 mM cAMP, a known co-factor for CRP binding, was used for our initial mass spectrometry runs of M9-glucose, M9-arabinose, stationary phase, and high osmolarity growth conditions, where we expected there to be annotated (e.g. *mglB* and *araB*) or predicted (e.g. *yadI*) binding sites of CRP. Since no significant enrichment for CRP was found for any of these runs, cAMP was not added to lysates for the other growth conditions. For high osmolarity, we based the concentration of supplemented salts on Figure 3 of Shabala et.al [128], which show measurements of intracellular salt concentrations for growth in external media of varying NaCl concentrations.

Time	Duration	Flow (nl/min)	%B
0:00	0:00	300	2
7:30	7:30	300	6
90:00	72:00	300	25
120:00	30:00	300	40
121:00	1:00	300	98
130:00	9:00	300	98

Table S3. Liquid chromatography gradient parameters for mass spectrometry. Mobile Phase A contains 0.2% formic acid, 2% acetonitrile, and 97.8% water. Mobile Phase B contains 0.2% formic acid, 80% acetonitrile, and 19.8% water.

**Global Settings**	
Ion source type	NSI
Spray voltage	1500 V
Ion transfer tube temperature	275 C
Polarity	Positive
**MS1 Scan Settings**	
Resolution	120000
Normalized AGC target	250
Maximum IT	50 msec
Scan range	375-1600 m/z
**MS2 Scan Settings**	
Resolution	50000
Normalized AGC target	Standard
Maximum IT	Dynamic
Loop time	3 sec
Isolation window	0.7 m/z
NCE	35
Spectrum data type	Centroid
Fixed first mass	110 Z

Table S4. Mass spectrometry scan settings for TMT samples

**Sequest HT Settings**	
Enzyme name	Trypsin (Full)
Max. missed cleavage	2
Min. peptide length	6
Max. peptide length	144
Precursor mass tolerance	10 ppm
Fragment mass tolerance	0.02 Da
Max. equal modification	3
Dynamic modification	Oxidation/ +15.995 Da (M)
Dynamic modification (peptide terminus)	Acetyl/ + 42.011 Da (N-Terminal)
Dynamic modification (peptide terminus)	Met-loss/ - 131.040 Da (M)
Dynamic modification (peptide terminus)	Met-loss+Acetyl/ - 89.030 Da (M)
Static modification (peptide terminus)	TMTpro/ + 304.027 Da (N-Terminal)
Static modification	TMTpro/ + 304.027 Da (N-Terminal)
Static modification	Carbamidomethyl/ + 57.021 Da (C)
**Percolator**	
Target/Decoy selection	Concatenated
Validation based on	q-Value
Target FDR (Strict)	0.01
Target FDR (Relaxed)	0.05

Table S5. Search parameters for Protein Discoverer 2.5

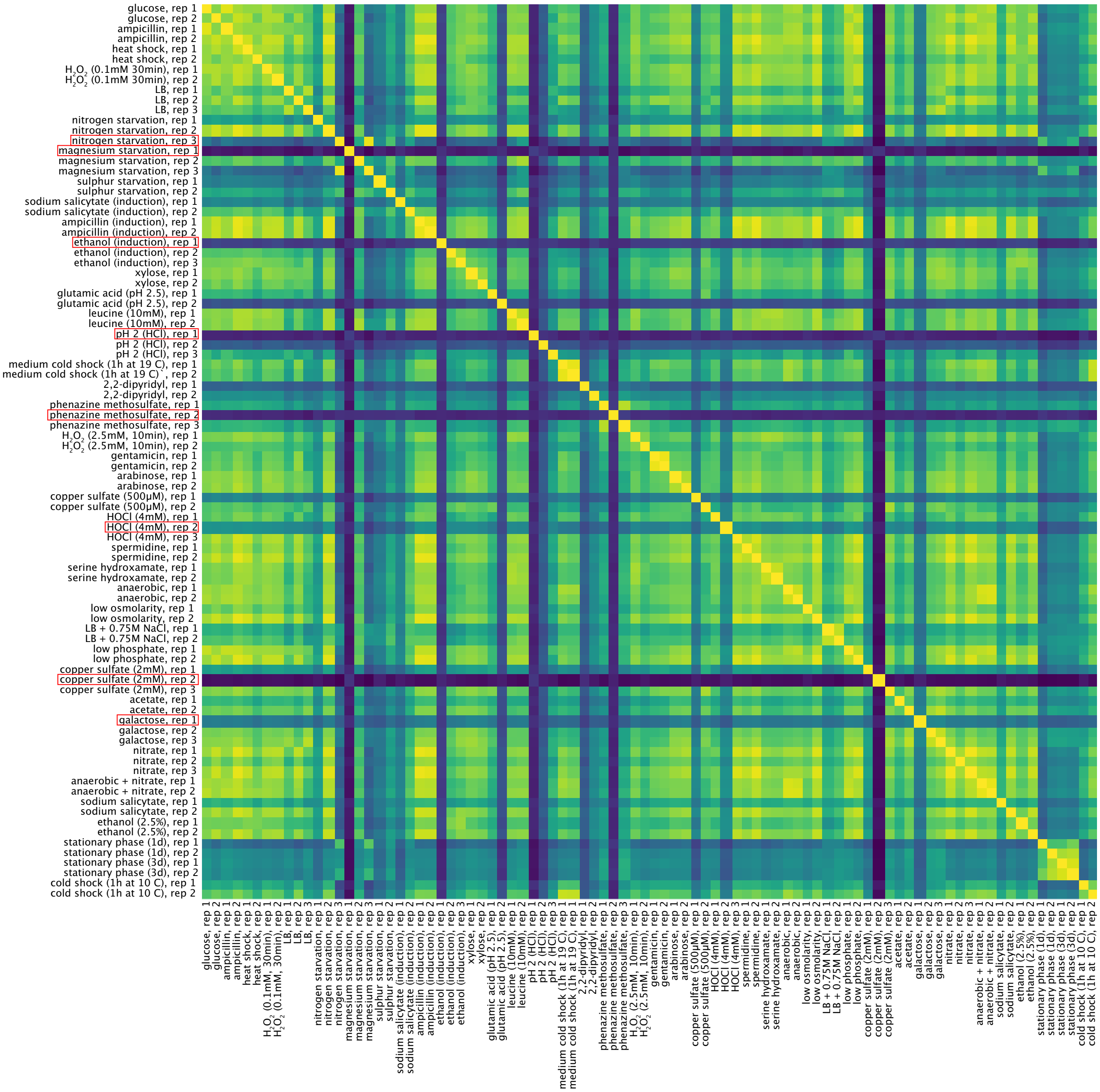

Figure S5. Correlation between experiments. The pearson correlation coefficient computed between all pairs of experiments. Experiments removed from further analysis are boxed in red.

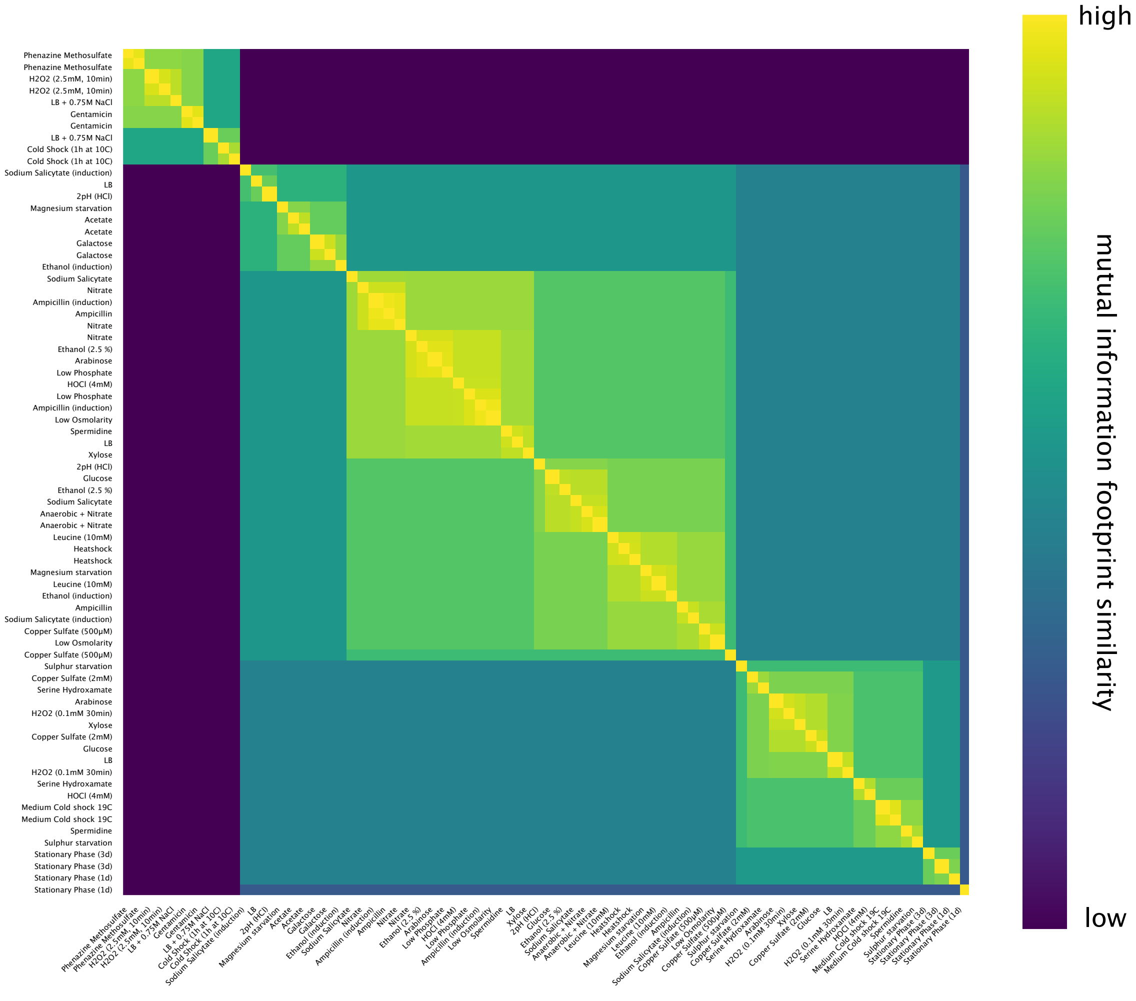

Figure S6. The fully annotated clustering figure of Figure 6. Conditions with multiple annotations are different replicates for the same condition.

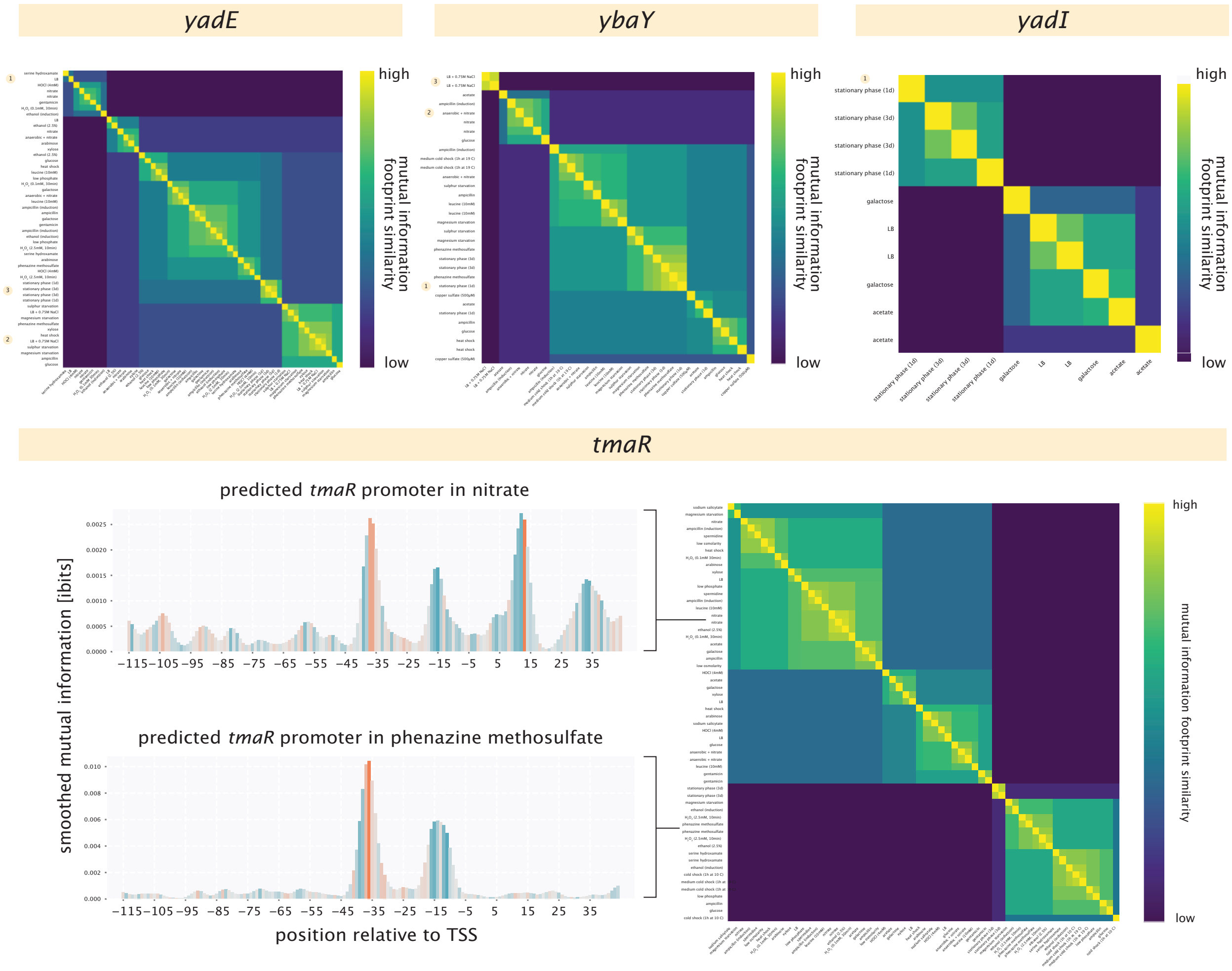

Figure S7. Clustering of conditions for ***yadE*****,** ***ybaY*****,** ***yadI*** **and** ***tmaR*****.** Results of hierarchical clustering are displayed. Footprints are shown for the genes in the upper panel in Figure 8. Numbers indicate which that there is a footprint shown for this specific condition.

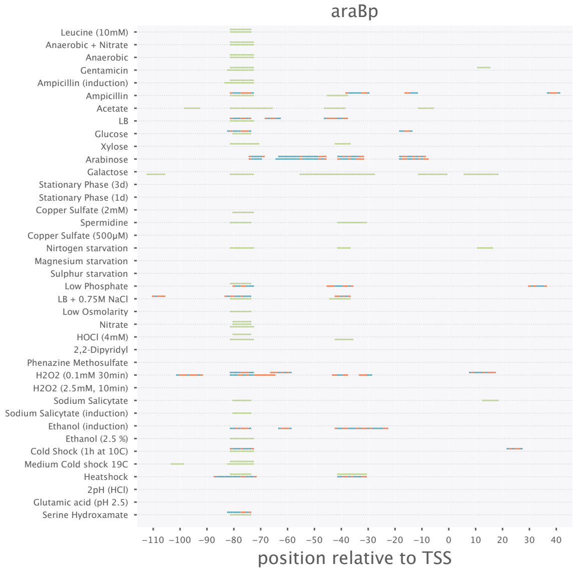

Figure S8. Inferred putative binding sites for the *araB* promoter.

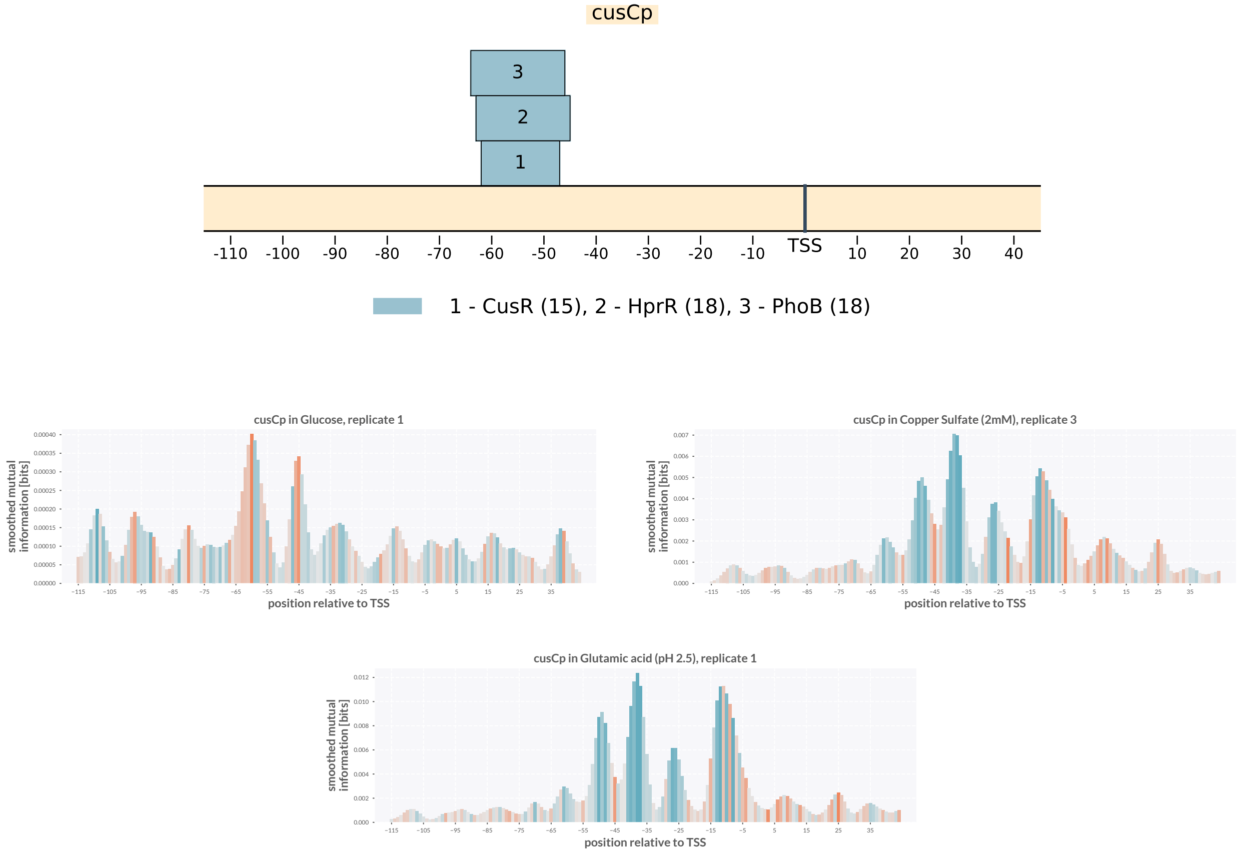

Figure S9. Annotated regulation and information footprints for the *cusCFBA* promoter. Footprints are shown for growth in minimal media with glucose, when induced with coppers sulfate and for shock in minimal media with glucose at 2.5 pH and with 1 mM of glutamic acid.

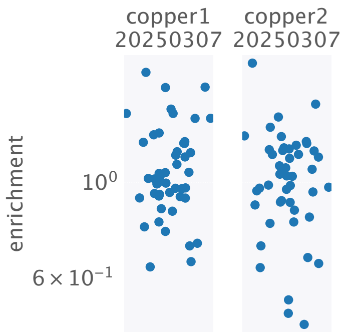

Figure S10. Mass-spec results for ***cusC*****.** Enrichment for transcription factors is shown for mass-spec experiments where lysates from cells were used that were induced with 2 mM of copper sulfate for 1h after reaching exponential phase.

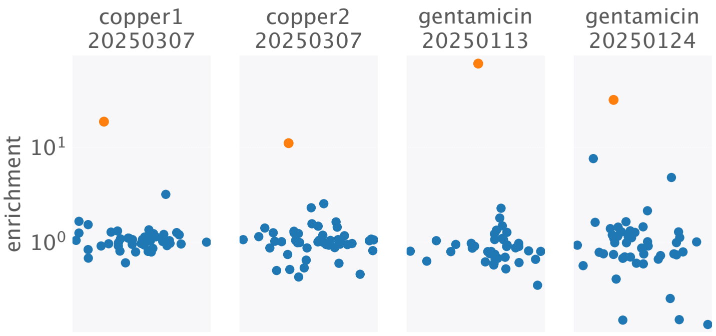

Figure S11. Mass-spec results for ***cpxR*****.** Cells were grown and induced either with gentamicin or copper sulfate. Highlighted is CpxR in orange.

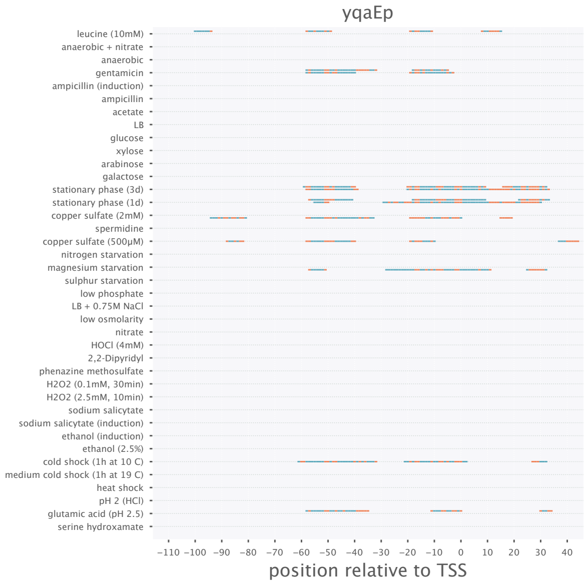

Figure S12. Inferred putative binding sites for the $yqaE$ promoter.

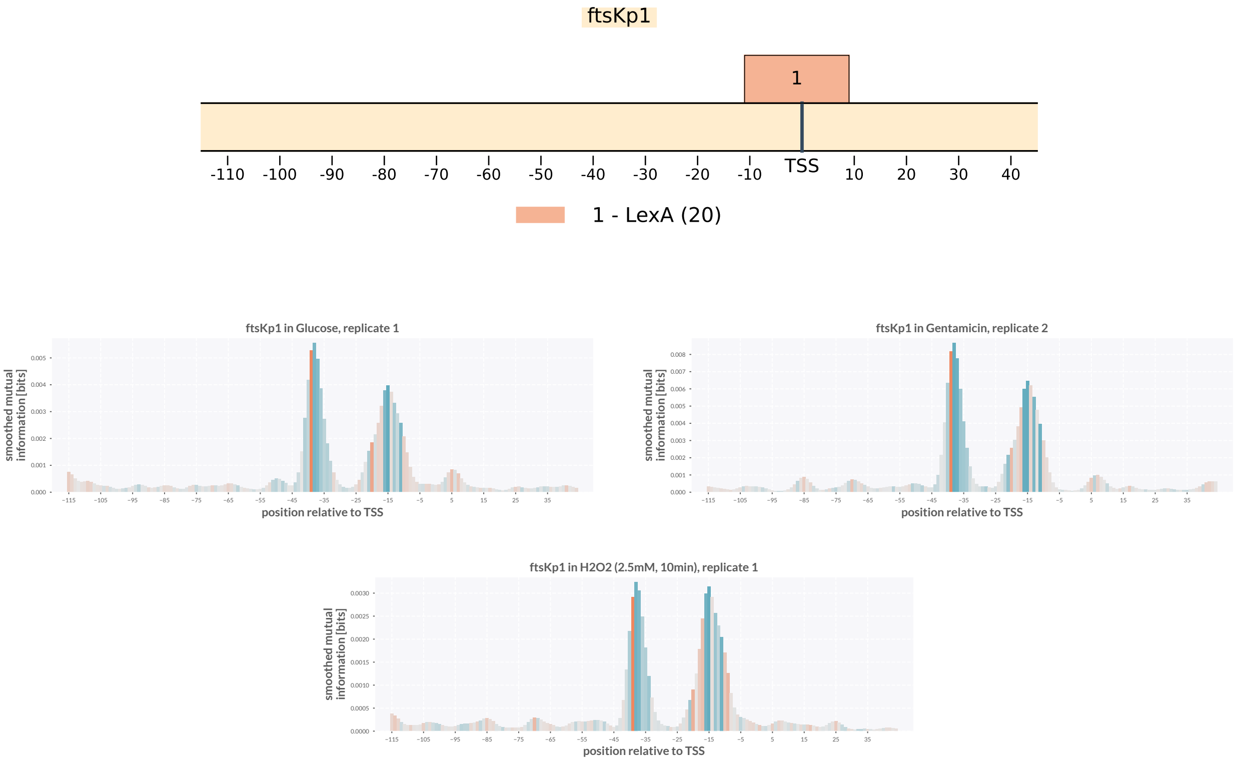

Figure S13. Annotated regulation and information footprints for *ftsK* promoter *ftsKp1*. Footprints are shown for growth in minimal media with glucose, and induction with gentamicin or hydrogen peroxide.

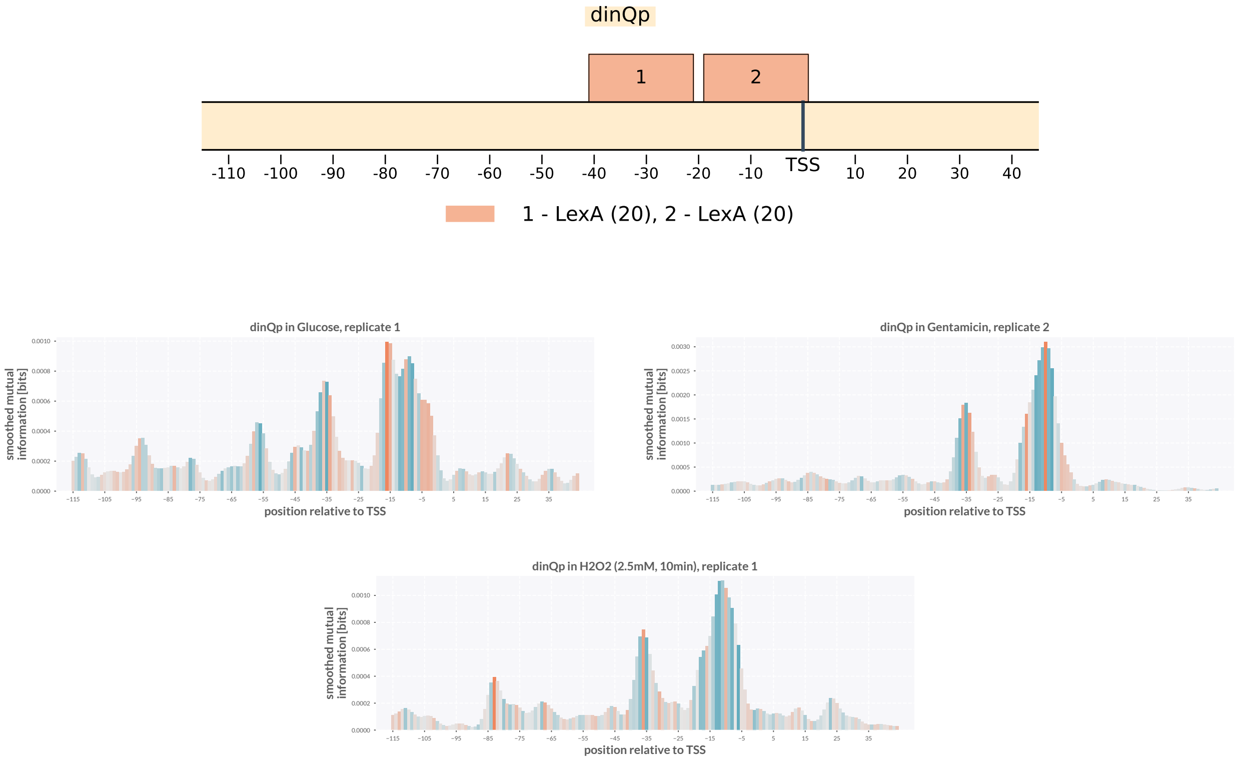

Figure S14. Information footprints for the *dinQ* promoter. Footprints are shown for growth in minimal media with glucose, induction with gentamicin and induction with hydrogen peroxide.

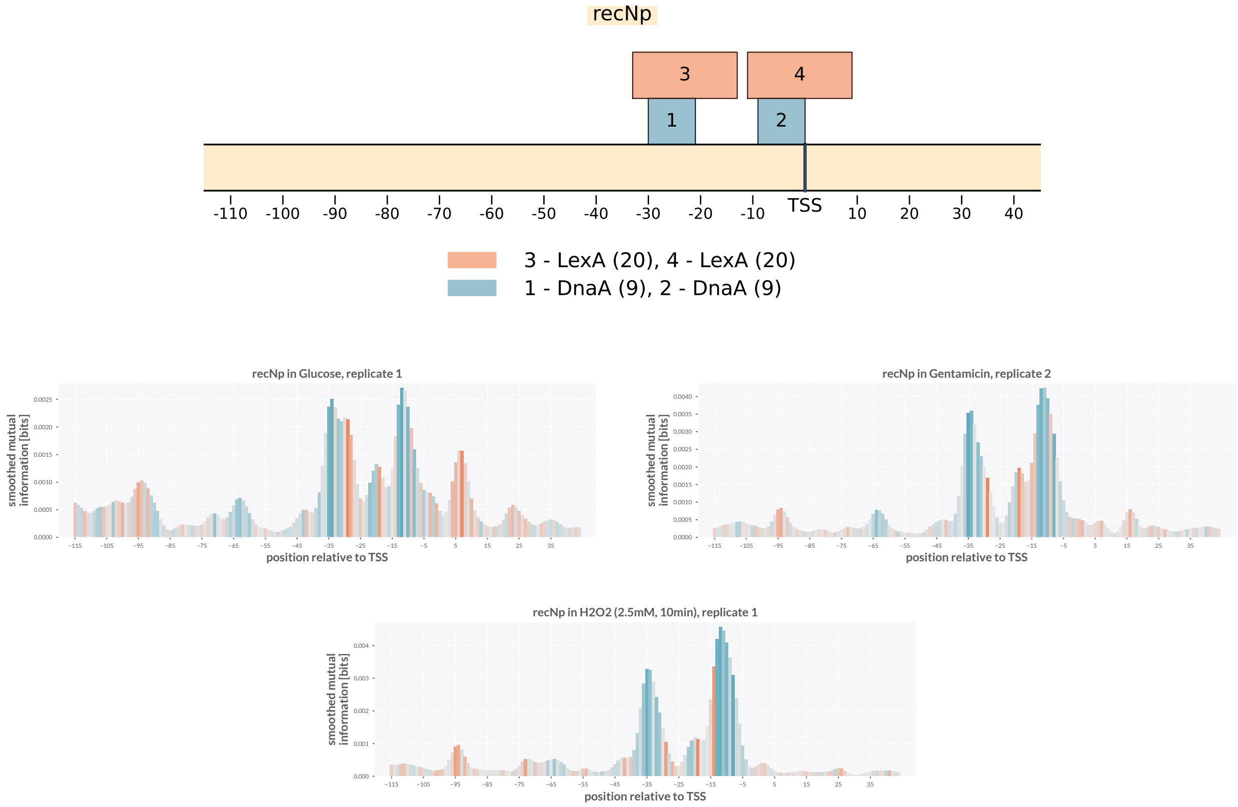

Figure S15. Annotated regulation and information footprints for the *recN* promoter. Footprints are shown for growth in minimal media with glucose, induction with gentamicin, and induction with hydrogen peroxide.

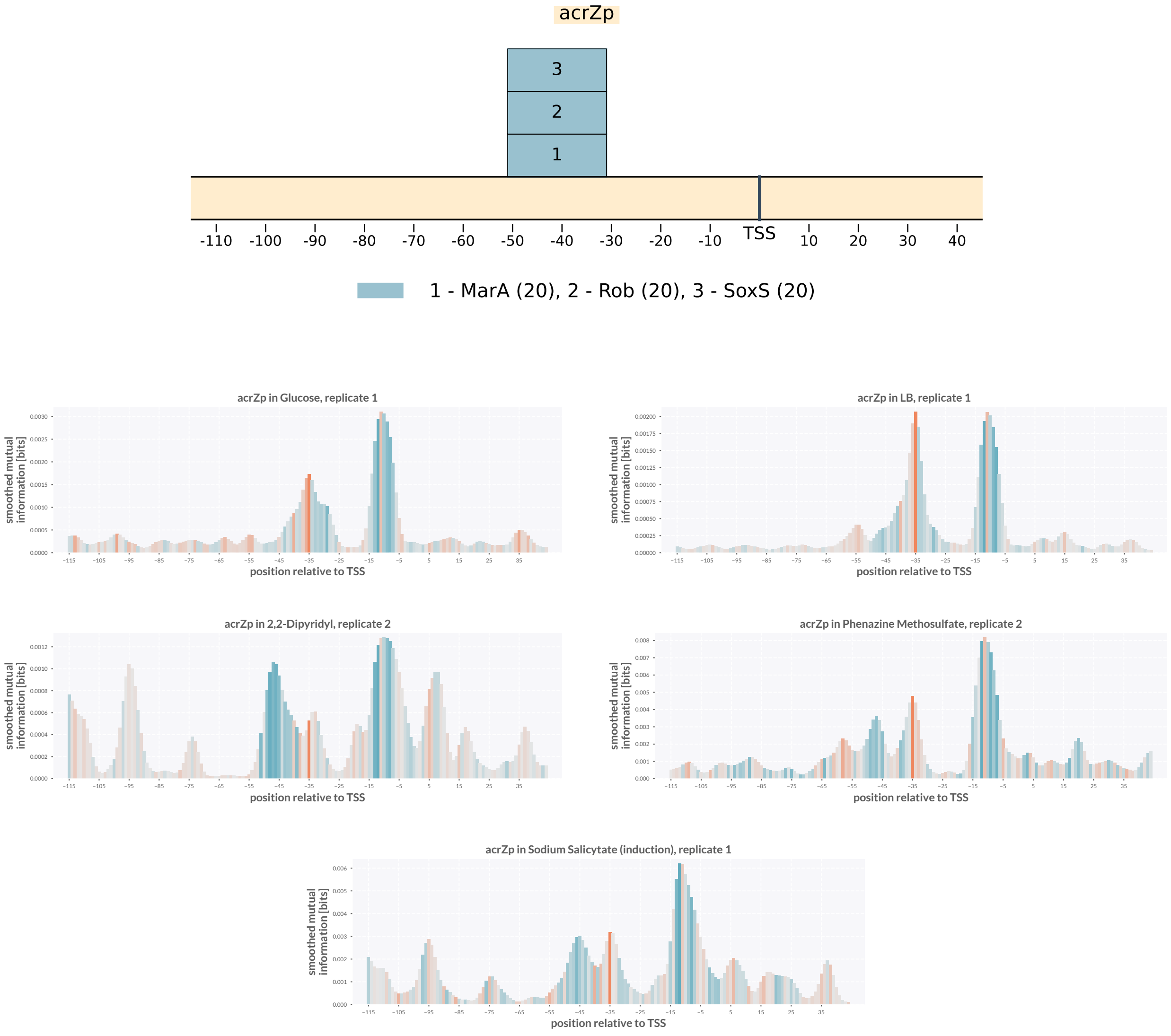

Figure S16. Information footprints for the *acrZ* promoter. Footprints are shown for minimal media with glucose, LB, induction with 2,2-dipyridyl, induction with phenazine methosulfate, and induction with sodium salicylate.

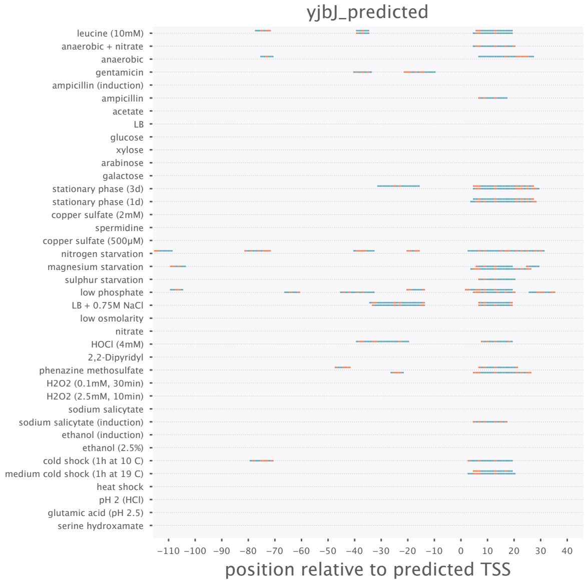

Figure S17. Inferred putative binding sites for the predicted promoter for ***yjbJ*****.**

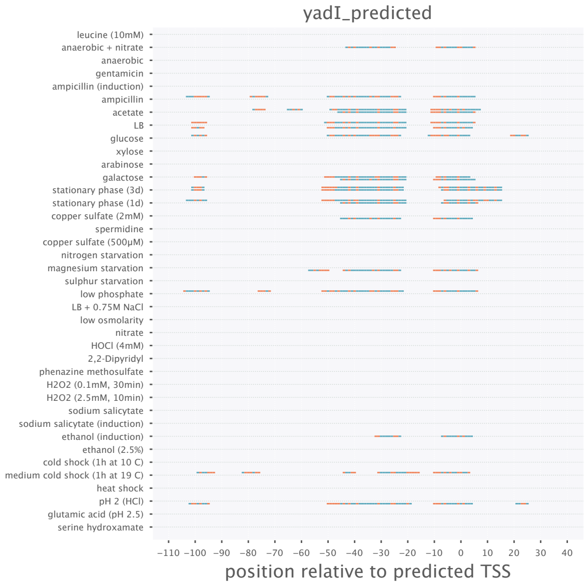

Figure S18. Inferred putative binding sites for the predicted ***yadI*** **promoter.**

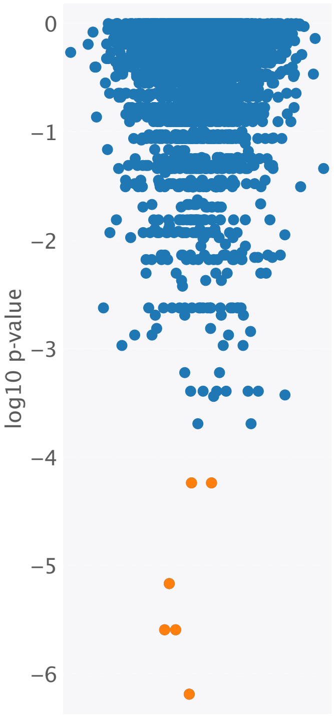

Figure S19. P-values for known binding sites matching with the putative activator site of *yadI*. The top six hits for CRP are highlighted in orange.

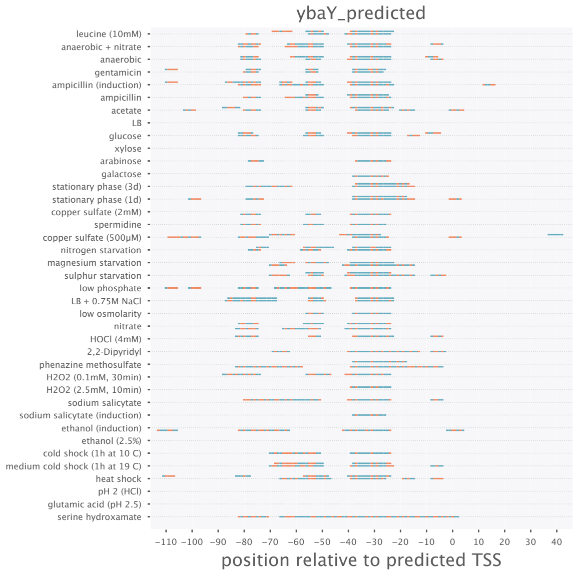

Figure S20. Inferred putative binding sites for *ybaY* promoters.

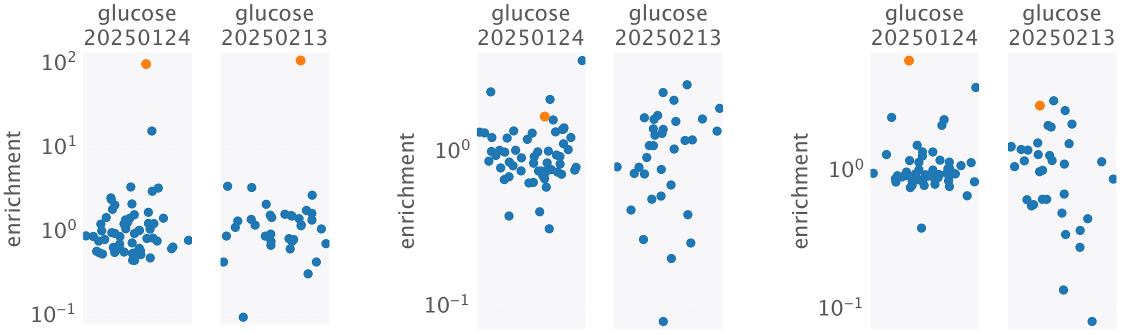

Figure S21. Mass-spec results for *ybiY-ybiW*. Left to right: *ybiY1* (covering -50 to +30), *ybiY2* (-50 to -5), *ybiY3* (-5 to 30). Highlighted in orange is the transcription factor YciT.

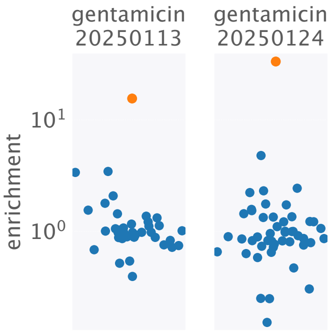

Figure S22. Mass-spec results for *intE-xisE-ymfH*. Highlighted in orange is the transcription factor YhaJ.

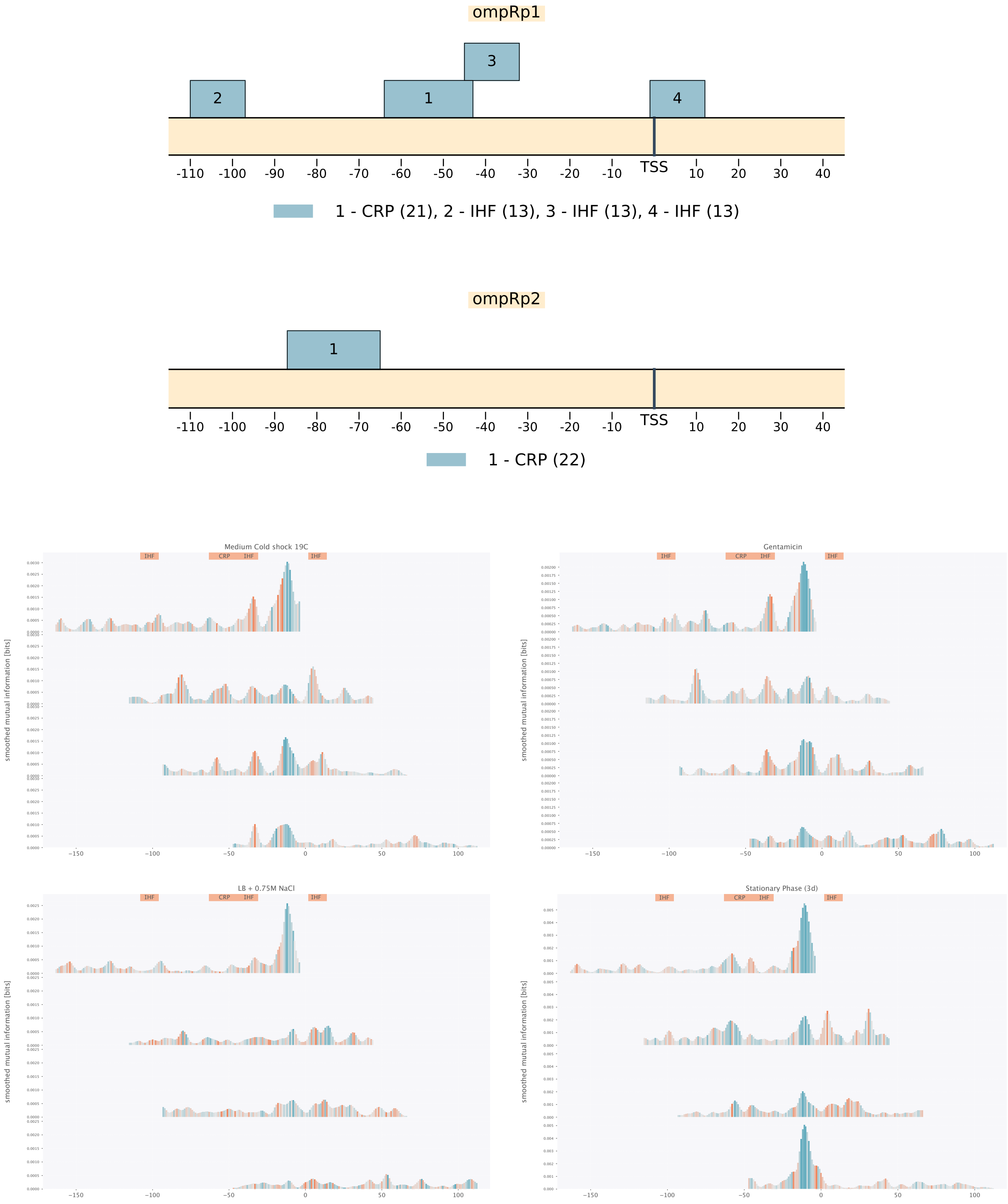

Figure S23. Annotated regulation and information footprints for the *ompR* promoters. Footprints are shown for all four promoters, which are aligned such that 0 is the location of the transcription start site off *ompRp1*. Conditions shown are cold shock at 19C, induction with gentamicin, shock in LB with high salt concentration and stationary phase after 72h.

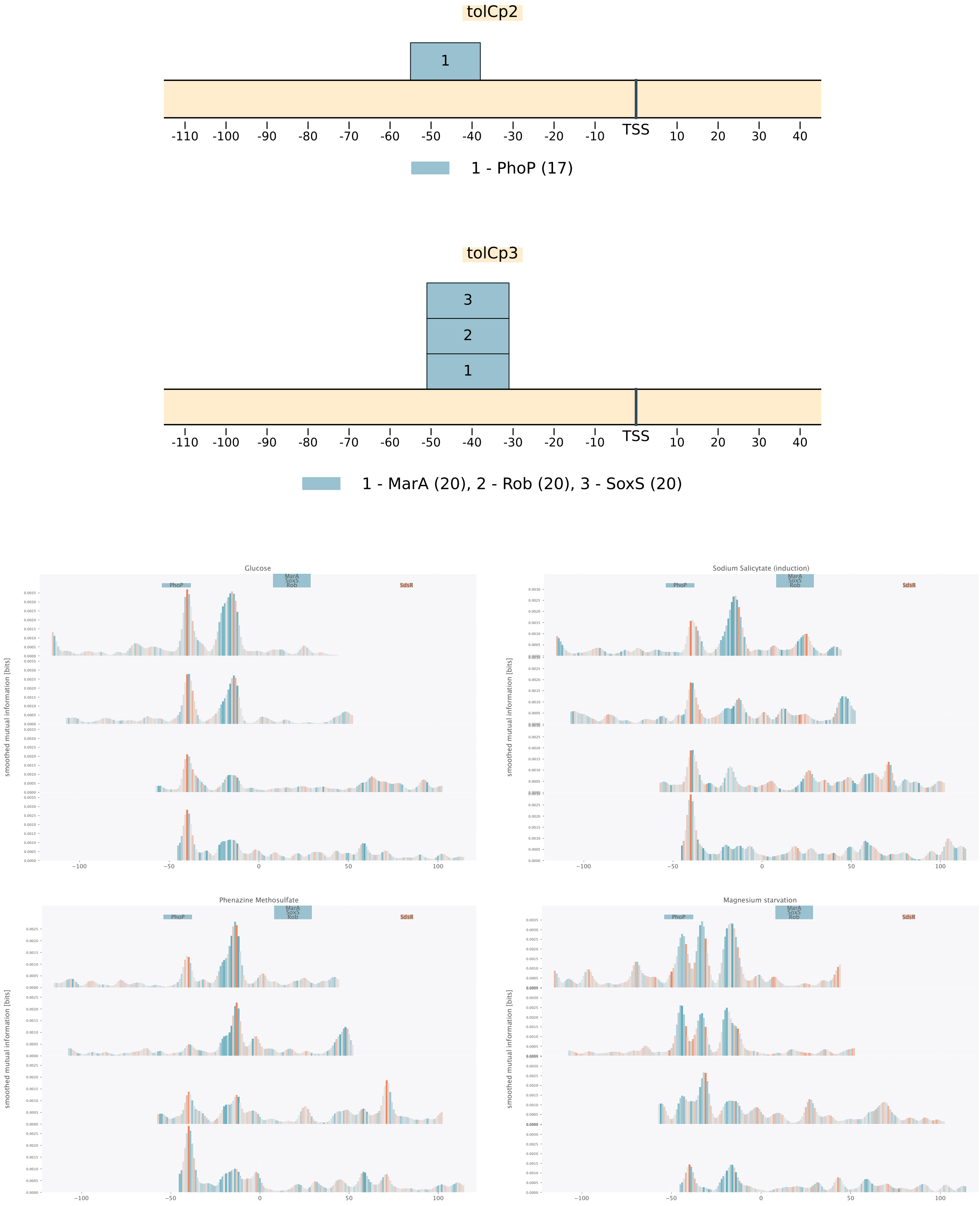

Figure S24. Annotated regulation and information footprints for the *tolC* promoters. Footprints are shown for all four promoters, which are aligned such that 0 is the location of the transcription start site off *tolCp1*. Conditions shown are growth in minimal media with glucose, induction with sodium salicylate or phenazine methosulfate and for magensium starvation.

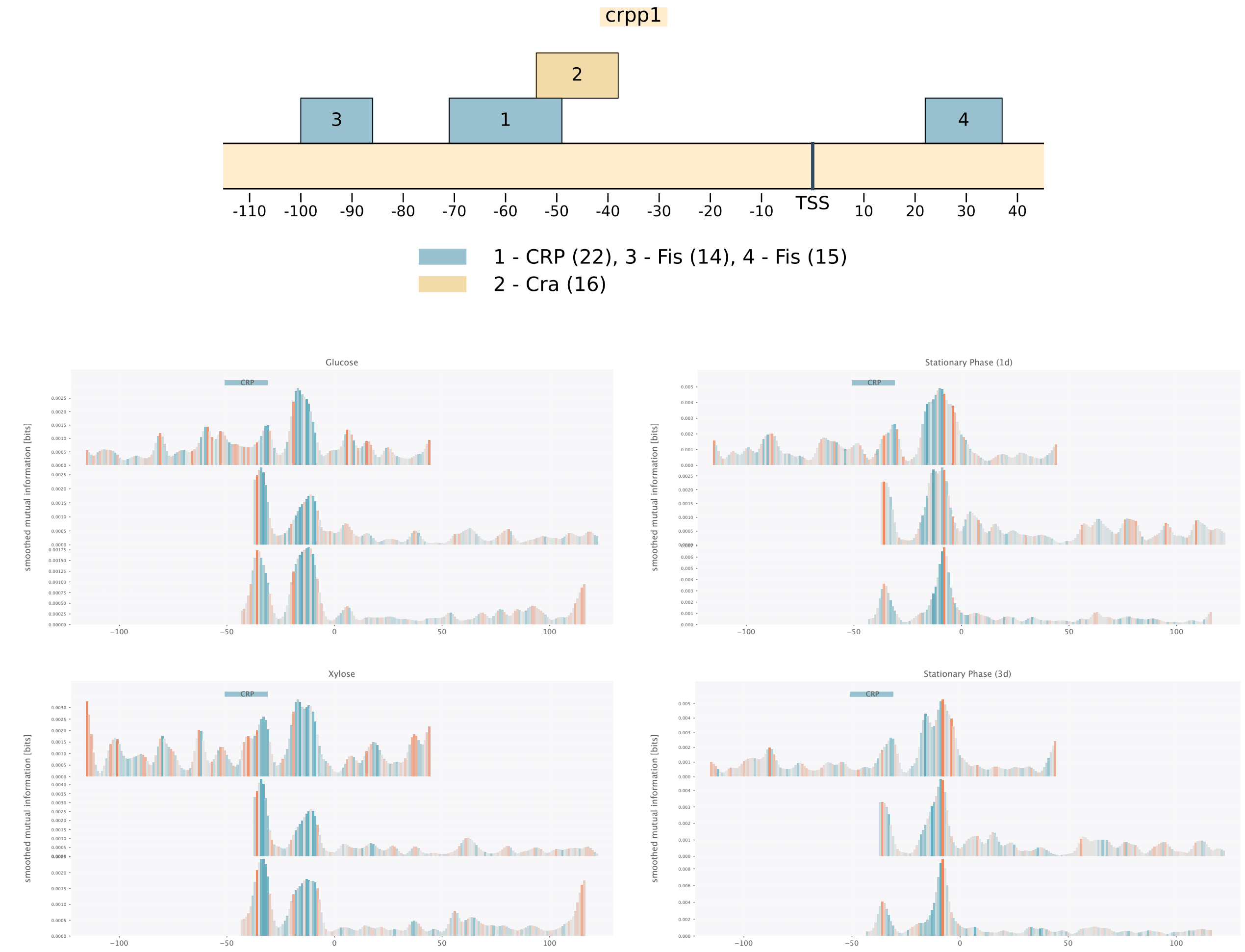

Figure S25. Annotated regulation and information footprints for the *crp* promoters. Footprints are shown for all three annotated *crp* promoters (top *crpp1*, middle *crpp2*, bottom *crpp3*) in minimal media with glucose or xylose as carbon sourses, and for stationary phase after 24h or 72h.

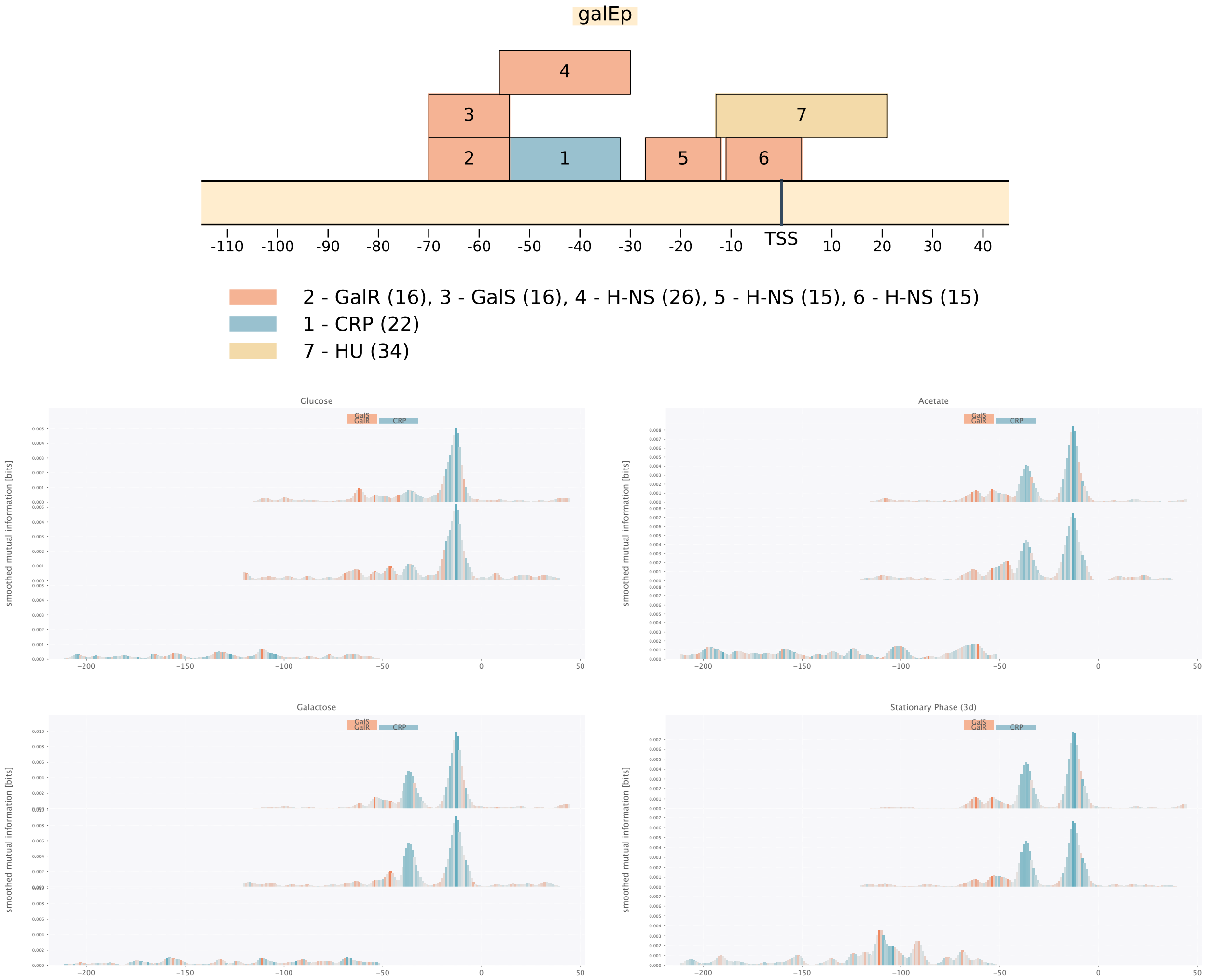

Figure S26. Annotated regulation and information footprints for the *galEp* promoters. Footprints are shown for all three promoters, which are aligned such that 0 is the location of the transcription start site off *galEp1*. Conditions shown are growth in minimal media with glucose, acetate or galactose as carbon source and for stationary phase after 72h.

Supplemental References

105. Ptashne, M. *A genetic switch: phage lambda revisited* 3rd (Cold Spring Harbor Laboratory Press, Cold Spring Harbor, N.Y., 2004).

106. Oehler, S., Eismann, E. R., Kramer, H. & Müller-Hill, B. The three operators of the *lac* operon cooperate in repression. *EMBO J* **9,** 973–9 (1990).

107. Oehler, S., Amouyal, M., Kolkhof, P., von Wilcken-Bergmann, B. & Müller-Hill, B. Quality and position of the three *lac* operators of *E. coli* define efficiency of repression. *EMBO J* **13,** 3348–55 (1994).

108. Muller, J., Oehler, S. & Muller-Hill, B. Repression of *lac* promoter as a function of distance, phase and quality of an auxiliary *lac* operator. *J Mol Biol* **257,** 21–9 (1996).

109. Kuhlman, T., Zhang, Z., Jr., M. H. S. & Hwa, T. Combinatorial transcriptional control of the lactose operon of *Escherichia coli*. *Proc Natl Acad Sci U S A* **104,** 6043–8 (2007).

110. Lewis, D. E. & Adhya, S. *In vitro* repression of the gal promoters by GalR and HU depends on the proper helical phasing of the two operators. *J Biol Chem* **277,** 2498–504 (2002).

111. Lee, D. H. & Schleif, R. F. In vivo DNA loops in araCBAD: size limits and helical repeat. *Proc Natl Acad Sci U S A* **86,** 476–80 (1989).

112. Lee, D. H., Huo, L. & Schleif, R. Repression of the araBAD promoter from araO1. *J Mol Biol* **224,** 335–41 (1992).

113. Ellington, A. D. & Szostak, J. W. In vitro selection of RNA molecules that bind specific ligands. *nature* **346,** 818–822 (1990).

114. Berger, M. F. & Bulyk, M. L. Universal protein-binding microarrays for the comprehensive characterization of the DNA-binding specificities of transcription factors. *Nature protocols* **4,** 393–411 (2009).

115. Weirauch, M. T. *et al.* Determination and inference of eukaryotic transcription factor sequence specificity. *Cell* **158,** 1431–1443 (2014).

116. O'Malley, R. C., Huang, S.-s. C., Song, L., Lewsey, M. G., Bartlett, A., Nery, J. R., Galli, M., Gallavotti, A. & Ecker, J. R. Cistrome and epicistrome features shape the regulatory DNA landscape. *Cell* **165,** 1280–1292 (2016).

117. Bartlett, A., O'Malley, R. C., Huang, S.-s. C., Galli, M., Nery, J. R., Gallavotti, A. & Ecker, J. R. Mapping genome-wide transcription-factor binding sites using DAP-seq. *Nature protocols* **12,** 1659–1672 (2017).

118. Rhee, H. S. & Pugh, B. F. ChIP-exo method for identifying genomic location of DNA-binding proteins with near-single-nucleotide accuracy. *Current protocols in molecular biology* **100,** 21–24 (2012).

119. Peano, C. *et al.* Characterization of the *Escherichia coli* σ S core regulon by chromatin immunoprecipitation-sequencing (ChIP-seq) analysis. *Scientific reports* **5,** 10469 (2015).

120. Fitzgerald, D. M., Stringer, A. M., Smith, C., Lapierre, P. & Wade, J. T. Genome-wide mapping of the *Escherichia coli* PhoB regulon reveals many transcriptionally inert, intragenic binding sites. *MBio* **14,** e02535–22 (2023).

121. Lioy, V. S., Cournac, A., Marbouty, M., Duigou, S., Mozziconacci, J., Espéli, O., Boccard, F. & Koszul, R. Multiscale structuring of the E. coli chromosome by nucleoid-associated and condensin proteins. *Cell* **172,** 771–783 (2018).

122. Grainger, D. C., Hurd, D., Harrison, M., Holdstock, J. & Busby, S. J. Studies of the distribution of *Escherichia coli* cAMP-receptor protein and RNA polymerase along the E. coli chromosome. *Proceedings of the National Academy of Sciences* **102,** 17693–17698 (2005).

123. Chung, D., Park, D., Myers, K., Grass, J., Kiley, P., Landick, R. & Keleş, S. dPeak: high resolution identification of transcription factor binding sites from PET and SET ChIP-Seq data. *PLoS computational biology* **9,** e1003246 (2013).

124. Lally, P. *et al.* Predictive Biophysical Neural Network Modeling of a Compendium of in vivo Transcription Factor DNA Binding Profiles for *Escherichia coli. bioRxiv* (2024).

125. Freddolino, P. L., Amemiya, H. M., Goss, T. J. & Tavazoie, S. Dynamic landscape of protein occupancy across the *Escherichia coli* chromosome. *PLoS Biology* **19,** e3001306 (2021).

126. Kosuri, S., Goodman, D. B., Cambray, G., Mutalik, V. K., Gao, Y., Arkin, A. P., Endy, D. & Church, G. M. Composability of regulatory sequences controlling transcription and translation in *Escherichia coli. Proceedings of the National Academy of Sciences* **110,** 14024–14029 (2013).

127. Salgado, H. *et al.* RegulonDB v12. 0: a comprehensive resource of transcriptional regulation in *Escherichia coli* K-12. *Nucleic Acids Research* **52,** D255–D264 (2024).

128. Shabala, L., Bowman, J., Brown, J., Ross, T., McMeekin, T. & Shabala, S. Ion transport and osmotic adjustment in *Escherichia coli* in response to ionic and non-ionic osmotica. *Environmental microbiology* **11,** 137–148 (2009).
